# The Mitotic Apparatus and Kinetochores in Microcephaly and Neurodevelopmental Diseases

**DOI:** 10.3390/cells9010049

**Published:** 2019-12-24

**Authors:** Francesca Degrassi, Michela Damizia, Patrizia Lavia

**Affiliations:** 1 IBPM Institute of Molecular Biology and Pathology, CNR Consiglio Nazionale delle Ricerche, c/o Department of Biology and Biotechnology “Charles Darwin”, Sapienza University of Rome, 00185 Roma, Italy; 2 Department of Biology and Biotechnology “Charles Darwin”, Sapienza University of Rome, 00185 Roma, Italy; michela.damizia@uniroma1.it

**Keywords:** microcephaly, mitotic apparatus, kinetochore, chromosome segregation, neural progenitors

## Abstract

Regulators of mitotic division, when dysfunctional or expressed in a deregulated manner (over- or underexpressed) in somatic cells, cause chromosome instability, which is a predisposing condition to cancer that is associated with unrestricted proliferation. Genes encoding mitotic regulators are growingly implicated in neurodevelopmental diseases. Here, we briefly summarize existing knowledge on how microcephaly-related mitotic genes operate in the control of chromosome segregation during mitosis in somatic cells, with a special focus on the role of kinetochore factors. Then, we review evidence implicating mitotic apparatus- and kinetochore-resident factors in the origin of congenital microcephaly. We discuss data emerging from these works, which suggest a critical role of correct mitotic division in controlling neuronal cell proliferation and shaping the architecture of the central nervous system.

## 1. Introduction

Regulators of the mitotic apparatus play key roles in orchestrating chromosome segregation. Therefore, their coordinated activity is critical to the transmission of genetic stability throughout cell generations. The onset of mutations, or regulatory alterations (under- or overexpression) of mitotic factors in differentiated somatic tissues often leads to abnormal mitosis, the loss of checkpoint function, and the generation of genetically unbalanced daughter cells [1]. This favors the onset of conditions that predispose cells to become genetically unstable, which is one of the best-established cancer hallmarks and is associated with unrestrained proliferation [1,2].

On the other hand, mitotic gene mutation or dysfunction occurring early during development can trigger the onset of syndromes that reflect a reduced number of neurons, e.g., microcephaly and related disorders [3,4,5]. It is actually emerging that a growing number of factors that regulate mitotic division have also roles in developmental neurological diseases, and those diseases often reflect insufficient cell proliferation and/or failure to replenish the neuronal stem cells that harbor genetic mutations in mitotic genes [3,6]. As a result, the central nervous system (CNS) remains underdeveloped. Thus, dysfunction of the same mitotic gene can yield either the overproliferation of genetically unbalanced cells in somatic tissues or insufficient proliferation in the developing brain.

Much of our understanding of mitotic control pathways comes from work with somatic cells, mostly from cultured cell lines, while tracking the actual journey of neuronal cell precursors in vivo has proven difficult: thus, translating our understanding of how the mitotic apparatus is regulated into the developing CNS is still presenting gaps, incomplete understandings, and pitfalls. Here, we will review the mitotic genes mutated in microcephaly with a special focus on the role of kinetochore factors that have been implicated in this disease. Then, we will confront some of the specificities and unresolved issues of mitotic dysfunction in the neuronal context.

## 2. Genes Implicated in Microcephaly

Primary microcephaly (MCPH) comprises a vast group of different disorders that are genetically heterogeneous yet all characterized by a severely reduced size of the head at birth: clinically, microcephaly is diagnosed as the presence of a head size three-fold standard deviations (SD) below that of the age- and sex-related mean [7]. MCPH is usually associated with a broad spectrum of other clinical traits and neurodevelopmental deficiencies that compromise, more or less severely, the quality of life and reflect severe defects during early neurogenesis [6,7].

To date, several unrelated *MCPH* loci, which is genetically associated with microcephaly, are identified based on the Online Mendelian Inheritance in Man (OMIM) database classification of *MCPH* genes (https://www.omim.org/). They currently sum up to 25 and are named *MCPH 1-25* according to the timing of their historical discovery. The growing use of whole genome sequencing techniques predicts that more loci will turn out to be involved [8]. Indeed, Table 1, which lists the OMIM-classified *MCPH* genes, is already incomplete in the light of the recent implication of further genes in similar developmental syndromes, as discussed below. Table 1 lists the “canonical” 25 *MCPH* loci identified thus far, together with relevant studies that have shed light on microcephaly-causing mutations; most studies present an in-depth genetic characterization of each individual locus. In building Table 1, we have attempted to integrate the information from single studies to extract general indications, and we have grouped the loci according to the intracellular localization and function of their encoded products. Some conclusions are astonishing:

1. Only one single type of microcephaly out of the 25 identified, i.e., *MCPH15* or *MFSD2A* (major facilitator superfamily domain containing 2A) [9], is associated with a mutation in a protein with a genuine neural-related function, i.e., a mutation in a transporter of lysophosphatidylcholine across the blood/brain barrier, which is directly required for proper brain growth.

2. Two loci encode structural components of global cellular machineries, which act respectively in the build-up of the Golgi (*MCPH19*, or *COPB2*) [10] and in the autophagic process (*MCPH18*, or *WDFY3*) [11], encoding a phosphatidylinositol 3-phosphate-binding protein.

3. The remaining 22 loci act in cell cycle functions or checkpoints. Although in some cases their mutations impinge on more than one specific process, it is clear that at least 20 loci encode factors that act either at the level of mitotic apparatus, or on mitotic chromosome organization, ultimately converging in the process of chromosome segregation.

The data in Table 1 [12,13,14,15,16,17,18,19,20,21,22,23,24,25,26,27,28,29,30,31,32,33,34,35,36,37,38,39,40,41,42,43,44,45,46,47,48,49,50,51,52,53,54,55] highlight a central role of mitosis in brain development and identify mitotic failure, more than defects in the function of genes regulating neural physiology, as subverting the neural developmental program. It would be out of context to summarize here the molecular details of mechanisms and factors governing the assembly and function of the mitotic apparatus, and we shall refer to recent extensive reviews on these subjects [12,13,14,15,16]. To the scope of this review, we wish to provide a brief outline of mitotic components known to harbor microcephaly-associated mutations: centrosomes, microtubule-associated factors, and kinetochores. We will focus on the latter, as kinetochore components are growingly implicated in MCPH through a variety of well-regulated mechanisms, yet no systematic integration of published data has as yet been attempted.

**Table 1:** Canonical microcephaly-associated gene products, localization, and function.

Gene Name ^a^	Protein Product	Protein Function	Refs
CHROMATIN- AND CHROMOSOME-ASSOCIATED PROTEINS			
*MCPH1*	Microcephalin, BRCT-repeat inhibitor of TERT expression 1	Regulates chromosome condensation; acts in intra-S and G2/M checkpoints	[17,18,19]
*MCPH10*	ZNF335	Chromatin remodeling complex regulating neuronal gene expression and cell fate	[20]
*MCPH11*	PHC1	Component of the Polycomb group (PcG) PRC1-like complex, regulating transcriptional repression and chromatin remodeling of developmental genes, e.g., Hox genes.	[21,22]
*MCPH21* *MCPH22* *MCPH23*	NCAPD2 (condensin I, subunit 1); NCAPD3 (condensin II, subunit D3); NCAPH (condensin I, subunit H)	Cooperate in compaction of interphase chromatin into mitotic chromosomes.	[23,24,25,26]
CENTROSOME DUPLICATION AND FUNCTION			
*MCPH3*	CDK5RAP2/CEP215, regulator of cyclin-dependent kinase 5	Centriole engagement and microtubule nucleation	[27,28,29]
*MCPH2*	WD40-Repeat Protein 62	Spindle organization, centrosome duplication	[30,31]
*MCPH8*	CEP135	Centriole biogenesis, duplication and elongation	[32,33]
*MCPH9*	CEP152	Centrosome duplication	[34,35]
*MCPH14*	SAS-6	Centrosome duplication, procentriole formation	[36,37]
*MCPH6*	CENPJ	Centrosome- and kinetochore-associated protein	[38,39,40]
*MCPH7*	STIL, SCL-interrupting locus protein	Centriole assembly and duplication	[41,42]
SPINDLE AND MICROTUBULE FUNCTION AND DYNAMICS			
*MCPH5*	ASPM	Mitotic spindle regulator	[43,44,45]
*MCPH17*	CIT, Citron Rho-interacting kinase	Cytokinesis, localizes the kinesin KIF14 to the central spindle and midbody	[46,47]
*MCPH20*	KIF14	Kinesin motor protein, acts at microtubules and midbody via interaction with CIT/MCPH17	[48,49]
*MCPH25*	MAP11 Microtubule associated protein 11	Mitotic spindle dynamics	[50]
KINETOCHORES			
*MCPH4*	CASC5/KNL1	Kinetochore assembly, microtubule attachments, SAC signaling	[51]
*MCPH13*	CENP-E	Stabilization of kinetochore-microtubule attachments, chromosome congression	[52,53]
CELL CYCLE TRANSITIONS			
*MCPH12*	CDK6	Cell cycle kinase, cell cycle entry	[54,55]
*MCPH16*	ANKLE2, Ankyrin repeat and LEM domain-containing protein 2	Regulates nuclear envelope reassembly at mitotic exit, promotes dephosphorylation of BAF/BANF1 possibly via PP2A	[56,57]
GLOBAL CELL ORGANIZATION AND FUNCTION			
*MCPH18*	WDFY3, WD repeat and FYVE domain-containing protein 3	Component of the autophagic machinery	[11,58]
*MCPH19*	COPB2, Coatomer subunit beta	Component of the Golgi and vesicular trafficking system	[10,59]
NEURAL CELL-SPECIFIC FUNCTION			
*MCPH15*	MFSD2A, Sodium-dependent lysophosphatidylcholine symporter 1	Expressed at the blood–brain barrier, transport of fatty acids	[9,60]

^a^ The list reports OMIM-listed, microcephaly-associated loci as per August 2019.

To date, a large proportion of well-characterized mitotic genes with well described implication in neurodevelopmental disorders involve essentially the organization/function of two cell structures with fundamental roles in cell division: the centrosomes and the spindle.

### 2.1. Centrosome Duplication and Function in Microcephaly

Table 1 shows that seven out of the 25 identified MCPH loci encode centrosomal regulators, and act at some stage of the process of centrosome duplication. In the well-described cases of centrosomal proteins mutated in MCPH2, MCPH3, MCPH8, MCPH9, and MCPH14, the common trait is that control of the centrosome duplication cycle, which must occur once and only once per cell cycle, is disrupted at some stage [31,33,35,36]. As extensively described in the literature, centrosome overduplication affects the formation of a proper bipolar mitotic spindle, and give rise to the formation of multipolar spindles during brain development [61,62]. In addition, multipolar spindles can also form after centriole splitting or fragmentation during centrosome migration after duplication to opposite poles of the cell, and/or in the early phase of mitotic microtubule nucleation, or as a result of excessive pressure from kinetochore-attached microtubules at metaphase [63]. In all of these cases, biophysical forces are imbalanced at the centrosome level without necessarily perturbing the centrosome duplication cycle [63]. Interesting in this respect is the implication in microcephaly, in addition to the OMIM-recognized genes, of the gene encoding RAN-binding protein 1 (RANBP1) [64], a regulator of the RAN nucleotide guanosine triphosphate (GTP) hydrolase (GTPase), that controls centrosome cohesion [65,66]. The formation of multipolar spindles, due to either altered centrosome duplication or fragmentation, impairs balanced chromosome segregation. Indeed, the mechanistic implication of centrosomal genes in microcephaly and related CNS syndromes is thought to reflect the occurrence of abnormal segregation along an aberrant axis of cell division. Spindle orientation is critical for the establishment of asymmetrical divisions that originate the stem versus proliferating the cell pool, which is typical of neural development, such that not only the genetic identity but also the functional and architectural properties of the nervous tissue are compromised. The underlying molecular pathways of such developmental abnormalities are extensively discussed in excellent reviews [62,67].

### 2.2. Spindle and Microtubule Function and Dynamics

Four identified genetic mutations (*MCPH5*, *MCPH17*, *MCPH20*, and *MCPH25*) listed in Table 1 involve genes regulating the dynamic function of microtubules. Among those, three (*MCPH5*, *MCPH17*, *MCPH20*) have clear roles in regulating the formation and function of the midbody. In addition to the genes listed in Table 1, citron kinase [47,68,69,70,71] and its cooperating factors [72] regulate the formation of the midbody at cytokinesis (reviewed in [73,74]). In those cases, the loss of viable cell products appears to reflect abnormalities in the process of cell separation. In certain types of MCPH, mutations impairing the function of the microtubule-binding kinesin KIF14 have also been identified [49]. The associated defects resemble those caused by citron kinase dysfunction, resulting in a failure of cell separation [49]. Interesting clues may also come from studies of CNS defects associated with viral infections. Indeed, defects in neural progenitor formation have been seen to occur at the level of the GTP-binding protein septins and hence in the proper unfolding of the cell cleavage process. Septins can be destroyed by the Zika virus-encoded proteases, with novel implications for the understanding of virally caused neurological diseases that can recapitulate neurodevelopmental defects [75].

## 3. The Rising Role of Kinetochores in Microcephaly

A growing number of kinetochore factors have been recently associated with cases of microcephaly, so that they can be considered newly rising players in developmental disorders [4,5,51,53,76,77]. Prior to examining the role of each of these factors, we will first briefly review kinetochore functions.

### 3.1. Roles of Kinetochores in Chromosome Segregation and Mitotic Fidelity

Accurate chromosome segregation at cell division is fundamental to maintain the species chromosome number from one cell generation to the next and prevent aneuploidy and chromosomal instability (CIN), which is commonly observed in cancer cells and is considered a driving force in the genesis and evolution of cancer [2,78].

The process of chromosome segregation relies on forces generated by the interaction of the kinetochore (the multiprotein structure that forms on centromeric chromatin) with spindle microtubules [16,79,80]. After the nuclear envelope breakdown, microtubules nucleated from spindle poles grow and shorten rapidly in the cellular space, randomly contacting kinetochores [12]. Although several mechanisms operate during spindle assembly to facilitate kinetochore–microtubule encounters [12], several erroneous kinetochore–microtubule interactions are formed, such as monotelic (one attached kinetochore and the sister kinetochore unattached), syntelic (both sister kinetochores attached to microtubules from the same pole), or merotelic (a single kinetochore attached to microtubules emanating from both spindle poles) interactions [81]. These improper attachments must be transformed before anaphase onset into a proper kinetochore–microtubule attachment, which is called amphitelic attachment (each sister kinetochore attached to a different spindle pole), to guarantee the correct chromosome segregation [81,82].

The accuracy of chromosome segregation is ensured by a control signal transduction pathway, the spindle assembly checkpoint (SAC), which prevents anaphase onset until all kinetochores are amphitelically or stably attached to the spindle [83,84,85]. Unattached or improperly attached kinetochores become the assembly platform for the mitotic checkpoint inhibitory complex (MCC) that inhibits the anaphase promoting complex/cyclosome (APC/C), to maintain high cyclin-dependent kinase 1 activity and centromeric cohesion [86]. When sister kinetochores achieve an amphitelic orientation, each kinetochore binds ≈20–25 microtubules to form a kinetochore fiber [87], so that sister kinetochores are under tension from forces exerted by microtubule-associated motor proteins. Full kinetochore occupancy and tension fulfill the requirements for SAC extinction, allowing anaphase onset by an APC-mediated resolution of centromeric cohesion and reduced cdk1 activity [83,86].

The fidelity of chromosome segregation is also ensured by an error correction pathway, which is a cell mechanism that promotes the release of incorrect kinetochore–microtubule attachments through the Aurora B-mediated phosphorylation of critical substrates, thus favoring the formation of amphitelic attachments [88,89]. Monotelic and syntelic orientations usually do not persist until anaphase, since their presence activates the SAC, which allows time for the correction mechanism to operate [89]. On the contrary, merotelic kinetochore orientations do not activate the SAC; therefore, there is a risky faulty interaction [81,82]. Thus, the persistence of incorrect kinetochore–microtubule attachments at anaphase generates aneuploidy and chromosome instability.

Our current understanding of the mitotic control pathways, as briefly described above, poses the kinetochore as a major player in the fidelity of mitotic cell division. In vertebrates, the kinetochore comprises more than 100 proteins that are organized into several subcomplexes [79]. The Constitutive Centromere Associated Network (CCAN) associates to the centromere region, as specified by the presence of nucleosomes containing the centromeric-specific histone H3 variant Centromere Protein-A (CENP-A) [90]. Association of the CCAN to the CENP-A nucleosomes throughout the cell cycle provides the structural determinants for the association of the other kinetochore complexes [91]. The KMN network is composed of the KLN1, MIS12, and NDC80 complexes and is recruited to the kinetochore at mitotic entry through its association to the CCAN [79,91]. Since the NDC80 complex directly interacts with microtubules, the kinetochore can be considered as a bridge connecting chromosomes and microtubules.

A plethora of other proteins are recruited to the kinetochore to participate in SAC activation and extinction and in the pathway correcting improper attachments. These include: mitotic arrest-deficient (MAD) and budding uninhibited by benzimidazoles (BUB) protein family members, microtubule-associated motor proteins, kinases, and phosphatases. Their functions have been discussed in depth in several excellent reviews, to which we address the reader for more information [79,83,85,92]. Beyond the well-established role of defective kinetochore functions in generating chromosome instability in cancer, recent studies have revealed mutations in kinetochore-associated genes in several microcephaly syndromes (reviewed in [77]). This has opened a new avenue of experimental work to understand the relationships between defective chromosome segregation and reduced brain growth that we intend to address.

### 3.2. Kinetochore Gene Mutations Associated with Microcephaly Syndromes

A crucial question emerging for microcephaly-driving mutations in kinetochore genes is why a mutation affecting such a ubiquitous process as chromosome segregation would generate a neuronal specific phenotype. To begin to shed light on this issue, in this section, we will examine the mitotic functions of kinetochore proteins implicated in MCPH phenotypes and the possible neuronal significance of their identified mutations.

#### 3.2.1. CASC5/KNL

The autosomal recessive primary microcephaly syndrome gene *MCPH4* was originally defined as a microcephaly linkage region on chromosome 15q15-21 [93], where a microcephaly-inducing mutation in the centrosomal *CEP152* gene was initially identified [35]. Within this linkage region, a mutation in *CASC5/KNL* was also found in three Moroccan families with *MCPH4* [51] and successively in one Algerian Family [94]. A second *CASC5* mutation was identified in a consanguineous Pakistani family afflicted with MCPH and a recent paper reported the first case of an infant, with microcephaly associated with the mutated *KNL1* gene, in an African American family [95,96].

*CASC5/KNL* encodes a large kinetochore protein (>250 kDa) required for kinetochore assembly, proper kinetochore microtubule interactions, and SAC signaling [85,86,97]. KNL1 associates to the CAAN complex via interaction with the MIS12 complex at its *C*-terminal domain and exhibits a direct microtubule-binding activity at the *N*-terminus. Accumulating evidence involves KNL1 in the kinetochore-based SAC activation. MPS1-dependent phosphorylation at the KNL1 *N*-terminus creates a docking site for BUB1 and its binding partner BUB3, which is essential to recruit further SAC proteins. KNL1 also activates the SAC through an additional interaction with the three-subunit Rod–Zwilch–ZW10 (RZZ) complex, which is required for MAD1 localization at unattached kinetochores [85,86].

**Figure 1:**
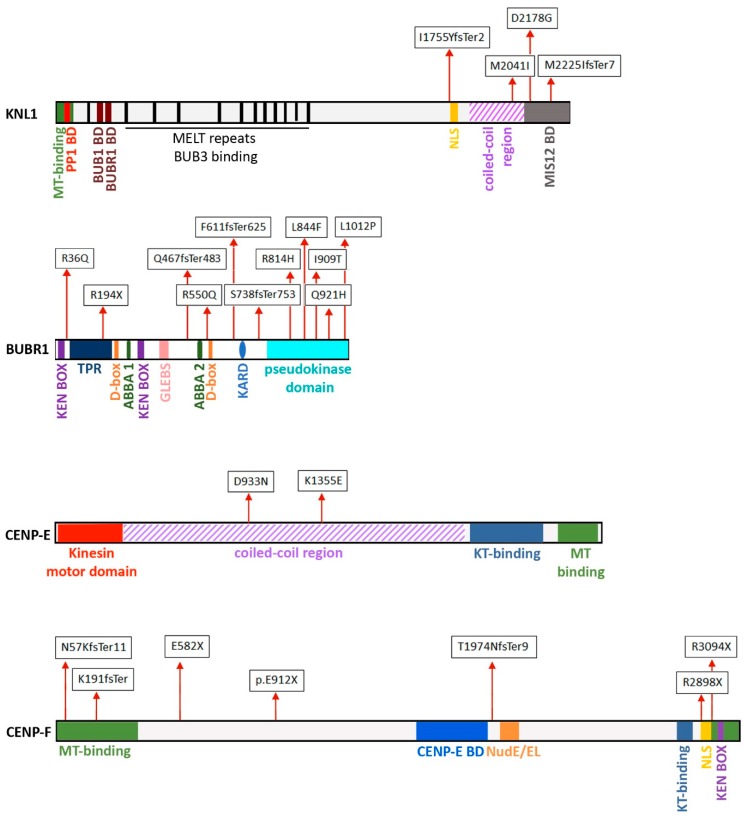
The maps depict relevant domains in kinetochore proteins associated with microcephaly when mutated. The sites of aminoacidic substitutions caused by microcephaly-associated mutations are boxed.

The first identified *CASC5/KNL* mutation in MCPH results in the skipping of exon 18 and causes protein truncation due to a frameshift that inserts a premature stop codon in exon 19 [51,94]. A second mutation induces the skipping of exon 25 [95], resulting in a frameshift mutation and production of a *C*-terminally truncated protein (see Figure 1). In both mutations, a reduced amount of the protein is produced, and, in addition, the exon 25 skipping mutation also caused an altered DNA damage response [95]. In both cases, as well as in the less characterized African American mutation, the mutant KNL1 lacks the MIS12 interaction region, suggesting that mutations in either the *N*-terminal or the central region might be lethal, whereas *C*-terminally localized mutations can give rise to congenital syndromes because they maintain a subset of protein interactions and sustain a residual protein activity.

Two recent papers have thoroughly investigated the question of the differential requirement for KNL domains and have provided new illuminating insights on the cause of brain-specific phenotypes. In the paper by Shi et al. [98], a conditional allele of KNL1 was produced to manipulate chromosome segregation in embryonic mouse brain and investigate the consequences of altered genome stability in vivo. A conditional deletion of *KNL1* using Cre recombination in cortical neural progenitor cells at E12.5 produced a 40% decrease in the cortical area at birth, which is consistent with the human microcephaly phenotype. The conditional deletion of *KNL1* in neural progenitor cells triggered a rapid P53-dependent apoptotic response and neural progenitor loss. This was associated with a lengthened S-phase duration in *KNL1*-deleted neural progenitors followed by anaphase chromosome damage in the form of lagging chromosomes and chromatin bridges, with the ensuing formation of interphase micronuclei [98]. Micronuclei have been shown to derive from segregation errors at mitosis promoting interphase DNA damage, which is a condition that has been shown to trigger P53 activation [99,100,101]. In the mutant developing brain, the elimination of abnormal karyotypes through apoptosis left only normal neural progenitors to continue dividing, resulting in cell loss and microcephaly. Conversely, the absence of p53 in a *KNL1*-null background resulted in preweaning lethality, indicating that the proliferation of genome-damaged cells did not allow organismal survival [98].

A definite hint on the brain-specific phenotype of *KNL* mutations was provided in a study that used the in vitro differentiation of human embryonic stem cells to monitor the effect of the mutation leading to exon 19 skipping on neuronal development [102]. The missense mutation was targeted by CRISPR/CAS9 editing to Human Embryonic Stem Cells (hESCs) that were then differentiated into neural progenitors. Mutant neural progenitors underwent a lengthening of mitosis that was associated with cytokinesis failure, cell death, and premature differentiation toward astrocytes and neurons. Finally, using 3D neural spheroids, the authors showed a reduction in the size of mutant spheroids, which could be attributed to reduced proliferation and premature differentiation of the neural progenitors [102]. Although differences in KNL1 protein levels had not been reported in patients fibroblasts or lymphocytes in previous work [51], decreased levels of KNL1 were found to be present in mutant neural progenitors, possibly reflecting neural-specific differences in *KNL1* mRNA processing. In contrast, mutant fibroblasts and mutant neural crest cells showed no reduction in KNL1 levels and no defective growth, demonstrating that the KNL1 mutation affects only neural progenitors, which was possibly due to the higher levels of splicing proteins that were identified in these cells [102].

#### 3.2.2. BUBR1 and other SAC Proteins

Mosaic variegated aneuploidy (MVA) is a rare recessive disorder characterized by microcephaly, growth, and mental retardation and by an increased risk of childhood malignancies such as Wilms tumor, rhabdomyosarcoma, and leukemia [4]. Lymphoblasts or fibroblasts from MVA patients present mosaicism for several trisomies and monosomies associated with premature sister chromatid separation, again stressing the connection between chromosome segregation defects associated to increased cancer risk and brain developmental defects. Monoallelic or biallelic mutations in the Budding Uninhibited by Benzimidazole 1B (*BUB1B*) gene, which encodes the BUB-related 1 (BUBR1) protein, have been found in several MVA families presenting microcephaly [4,76]. In both genetic conditions, a low BUBR1 protein expression was recorded, suggesting that BUBR1 partial deficiency is one of the important causes of MVA-related microcephaly [4,76,103].

BUBR1 is reported as a multi-domain pseudo-kinase required for SAC signaling by its association with CDC20 to form the APC/C inhibitory complex [86]. BUBR1 localizes to mitotic kinetochores through a BUB3–BUB1-mediated interaction with KNL1 [104] and binds CENP-E (centromere protein E) at the CENP-E unstructured coiled-coil region through its pseudo-kinase domain [105], promoting the formation of stable kinetochore microtubule interactions [106]. Furthermore, BUBR1 contributes to stabilize kinetochore–microtubule interactions by recruiting the PP2A-B56 phosphatase on its CARD domain [107]. Microcephaly-associated mutations are present in these domains (see Figure 1), suggesting that a defective structural organization of the mitotic kinetochores, mediated by lack of interactions of multifunctional proteins, could be at the heart of the microcephaly phenotype. Altogether, these findings suggest that the different microcephaly-promoting mutations converge interdependently to impair SAC function and microtubule attachment. The relevance of BUBR1-related impaired SAC function in neural development has been recently investigated through the conditional loss of BUBR1 in a mouse cerebral cortex [108]. In that work, BUBR1-deficient cortex displayed a strikingly reduced number of late-born neurons, recapitulating the microcephaly phenotype. Importantly, a decreased proportion of neural progenitors was found to be in mitosis, specifically in metaphase, after BUBR1 nearly complete loss and this mitotic defect was associated with massive apoptotic cell death [108]. These phenotypes suggest that a major effect of BUBR1 deficiency is to cause aberrant chromosome segregation due to SAC impairment and/or defective kinetochore–microtubule attachments. Unbalanced chromosome distribution, in turn, triggers the elimination of progenitors and neurons during neurogenesis, producing microcephaly.

CEP57 (centrosomal protein 57) is another gene mutated in MVA syndrome [109]. Although originally identified as a centrosomal protein, CEP57 was subsequently implicated in SAC activation: the protein localizes to the kinetochore through its MIS12 association and functions as a platform for targeting the MAD1–MAD2 complex to the kinetochore [110], again implicating SAC function in the neural phenotype. However, CEP57 mutant lymphoblasts from one MVA patient were recently found to be SAC-proficient, so that the relative contribution of centrosomal and kinetochore defects in CEP57-mutated microcephaly is still unclear [111].

Finally, very recent work has identified biallelic loss-of-function mutations in the *TRIP13* gene in three individuals displaying microcephaly, who developed Wilms tumor [111]. In mitosis, TRIP13 is a regulator of both SAC activation and extinction by its interaction with the crucial SAC effector MAD2. Accordingly, functional studies showed that *TRIP13*-mutant patient cells have a substantial SAC impairment, leading to a high rate of chromosome missegregation [111].

Overall, these different findings highlight the importance of the SAC and its partial inactivation in the pathogenesis of microcephaly and suggest that cell death processes activated by SAC disruption in neural stem cells might produce the low numbers and defective structural organization of neurons in microcephaly patients.

#### 3.2.3. CENP-E

CENP-E is a microtubule plus-end-directed kinesin required for proper kinetochore–microtubule attachments and for the congression of chromosomes to the spindle equator [112,113,114]. Mutations in *CENP-E* have been found in two siblings with microcephalic primordial dwarfism (MCPH13), i.e., microcephaly phenotypes associated with extremely short stature [53]. Compound heterozygous point mutations were identified in the two patients and were found to localize in the central coiled-coil region of the protein (see Figure 1), which is a region of structural flexibility that was found to be required for the dynamic interaction of CENP-E with microtubules [115]. The microcephaly-associated variant did not adversely impact on CENP-E expression or stability but CENP-E kinetochore localization at mitosis was severely impaired in patient lymphoblastoid cell lines [53]. This could affect SAC function and chromosome alignment because CENP-E-dependent autophosphorylation of the SAC kinase BUBR1 has been shown to be required for these processes [114]. The conditional expression of patient-derived CENP-E variants produced prometaphases with uncongressed polar chromosomes and multipolar mitotic spindles [53], which is in line with the mitotic defects observed after CENP-E depletion in experimental models [112,113,116]. Short-time ATR-dependent DNA damage response was unaltered in the conditional variants, but no further information on delayed DNA damage or cell death was reported [53]. Overall, impaired kinetochore–microtubule interaction stability is likely to be associated with CENP-E mutated microcephaly.

#### 3.2.4. CENP-F

CENP-F (centromere protein F) is a large centromere-associated protein that acts in cell division and morphogenesis and is also implicated in cilia formation in epithelial cells [5,117]. It is highly expressed from the G2 phase throughout the duration of mitosis up to mitotic exit. CENP-F harbors a nuclear localization signal (NLS) that enables it to accumulate at the nuclear envelope in the G2 phase of the cell cycle and to enter the nucleus via the association with transport receptors in import complexes [118,119]. When the nuclear envelope breaks down at prometaphase, CENP-F associates with the outer plate of the kinetochore until early anaphase [118]. That association constitutes a platform for other proteins implicated in the regulation of the stability of kinetochore interactions with the spindle microtubules. In late anaphase, CENP-F re-localizes to the spindle midzone and the intracellular bridge [118] and is eventually degraded after telophase; this requires two degradation sequences in the protein, particularly involving a region called the box KEN7, and the activity of the APC/C ubiquitin ligase [120]. The ability of CENP-F to interact with different structures is encoded in distinct protein domains (see the map in Figure 1): (i) a microtubule-binding domain, which is crucial to bind mitotic microtubules [121]; (ii) the NudE/EL binding domain, through which CENP-F interacts with dynein/dynactin and localizes the motor proteins at the nuclear envelope at the G2/M transition, via the interaction with the NUP133 nucleoporin (a component of the NUP107-160 subcomplex) [122,123,124], which is a critical localization because dynein is a crucial determinant of the nuclear envelope breakdown, and hence of the timing of mitotic entry; (iii) the kinetochore- binding and CENP-E-binding domains, both essential to localize CENP-F at kinetochores during mitosis [119,125]; (iiii) the *C*-terminal region of CENP-F, containing both the NLS and the KEN7 box. This domain can also interact with the *N*-terminal region of the NUP133 nucleoporin, and that interaction is necessary to localize the CENP-F/NUP133 complex at KTs [126]. The localization of CENP-F at the outer plate of kinetochores enables CENP-F to regulate the interactions between kinetochores and the mitotic spindle microtubules and thus underlies CENP-F function in chromosome segregation.

Growing studies implicate CENP-F in brain development. Several clinically relevant mutations of *CENP-F* have been identified in Stromme Syndrome, which is a severe disease affecting multiple systems that feature a ciliopathy and microcephaly [5,127,128]. Remarkably, these mutations fall in the *N*-terminal domain of CENP-F, which contains the MT-binding domain [5,127] or in the *C*-terminal domain, harboring both the NLS and the neighboring KEN degradation sequence [128]. Of those, one frameshift mutation produces a truncated protein devoid of the NLS signal [128], and two point mutations may affect the NLS function [5] (see the map in Figure 1).

Therefore, although diversified, a common trait of the mutations is that they would preclude the proper nuclear localization of CENP-F in late G2, which precedes its kinetochore association and the regulation of microtubule interactions at mitosis. In addition, the loss of the KEN sequence and failure to degrade CENP-F at the mitotic exit might also impinge on the proper reformation of nuclei in daughter cells from neuronal cell precursors.

#### 3.2.5. NUP133 and Other Nucleoporins

The NUP133 nucleoporin was originally identified in Xenopus egg extracts [129] and is a component of the nuclear pore ”basket”, facing the nucleoplasm. In late G2, just prior to mitotic entry, NUP133 is required to tether the dynein/dynactin complex to the nuclear envelope [124]. That complex has a crucial role for nuclear envelope–centrosome tethering, which is turn required both for microtubule-mediated tearing of the nuclear envelope at mitotic entry and for cortical migration of radial glial progenitors (RGPs), from which neurons, glia, and adult neuronal cell stem cells originate in the developing brain [130,131]. The dynein-recruiting NUP133 complex in RGPs includes the CENP-F and NudE/EL proteins and during the cell cycle, it acts in the G2 phase [130] sequentially to another complex also containing a nucleoporin, the RANBP2-bicaudal D (BICD2) complex, which also has roles in establishing the centrosome–nuclear envelope connection [131].

In early mitosis, after nuclear envelope disassembly, NUP133 moves, as part of the NUP107-160 nuclear pore subcomplex, and together they migrate to and regulate kinetochore attachments to microtubules. Therein, the recruitment of the NUP107-160 complex to kinetochores depends mainly on the NDC80 complex and CENP-F [126]. Mutations in the *NUP133* gene were identified in the Galloway–Mowat syndrome 8, which is a rare severe renal–neurological disease characterized by early-onset nephrotic syndrome associated with microcephaly [132]. The mutation resulted in aberrant splicing, with the insertion of a short intronic sequence between exons 25 and 26, and a significant reduction in NUP133 protein levels. In vitro functional studies in HeLa cells showed that the NUP133 mutant protein characterized in this syndrome fails to bind NUP107. Zebrafish knock-out (KO) models showed microcephaly with fewer neuronal cells, which was rescued by wild-type but not mutant NUP133 [132].

Interestingly, NUP107, another member of the NUP107-160 subcomplex, was also found to be mutated in a microcephaly form associated with nephrotic syndrome, which is similar to the Galloway–Mowat syndrome [133]. Patient-derived fibroblasts carrying that mutation display a lower number of nuclear pores, altered chromatin organization, and irregular perinuclear spaces of the nuclear envelope [133].

Together, these data implicate the NUP107-160 nuclear pore subcomplex, and particularly the NUP133 and NUP107 nucleoporins, in at least two critical processes required for the formation of viable neuronal cells during development: proper chromosome segregation via their kinetochore-regulatory activity, and apical migration of neuronal precursors in the architecture of the developing brain. It will be important to disentangle the contribution of each of these processes to brain development.

## 4. Forward Looks

Data emerging from studies on microcephaly-associated mutations indicate that specificities in the unfolding of the mitotic cell division program in early neuronal precursors can modulate their mitotic cell fate.

The data assessing the role of kinetochores in microcephaly in mouse models or 3D human brain organoids suggest that the response to mild segregation errors associated to defective kinetochore function may elicit the cell death of neural stem cells during their expansion phase (when symmetric divisions are more frequent), leading to a premature differentiation of neural precursors [98,108].

On the other hand, centrosome and SAC-mutated drosophila strains are reported to be more tolerant to aneuploidy in the developing brain than in other epithelial tissues and respond to aneuploidy by lengthening the G1 phase of the cell cycle and undergoing premature differentiation [134,135]. These findings suggest a species-specific regulation of the response to chromosome missegregation in the developing brain [62] that requires being addressed with ad hoc experimental models [136,137].

**Figure 2:**
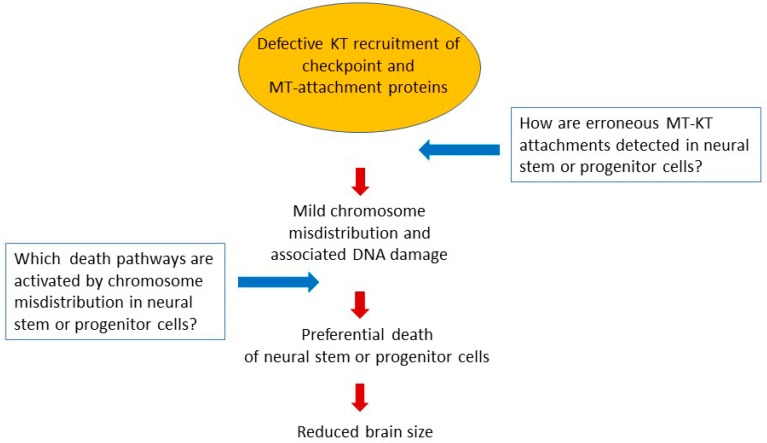
Hypothetical model and unresolved issues on the role of kinetochore dysfunction in microcephaly.

In conclusion, our current knowledge on mitosis dysfunction in neuronal development raises several issues that will have to be addressed in the near future (see Figure 2): under which circumstances do microcephaly reflect failures in asymmetric division, or aneuploidy clearance in embryonic brain stem cells? How does the mitotic checkpoint signal to the death apparatus in neural progenitors and in adult somatic cells? Is the capacity to detect and correct errors in microtubule–kinetochore attachment fully efficient in neural precursor types? How do these cells respond to uncorrected errors? A related unresolved issue concerns the role of motor proteins, including dynein and its interactors that recruit it to target structures, both in chromosome segregation via proper kinetochore function, and in the migration of neuronal precursors during the establishment of the brain architecture during development.

It may be hypothesized that control pathways for genetically unstable cells and their offspring are subjected to specific constraints during development of the central nervous system. Addressing these issues represents a novel forthcoming challenge, not only to unravel pathways implicated in these rare syndromes, but also to understand, from an ontogenetic point of view, the establishment of mitotic control pathways and the response to chromosome segregation errors in such critical cells as neural precursors.

## References

[1] Levine M.S., Holland A.J. (2018). The impact of mitotic errors on cell proliferation and tumorigenesis. Genes Dev..

[2] Bakhoum S.F., Landau D.A. (2017). Chromosomal instability as a driver of tumor heterogeneity and evolution. Cold Spring Harb. Perspect. Med..

[3] Jayaraman D., Bae B.-I., Walsh C.A. (2018). The Genetics of Primary Microcephaly. Annu. Rev. Genomics Hum. Genet..

[4] Hanks S., Coleman K., Reid S., Plaja A., Firth H., FitzPatrick D., Kidd A., Méhes K., Nash R., Robin N. (2004). Constitutional aneuploidy and cancer predisposition caused by biallelic mutations in BUB1B. Nat. Genet..

[5] Waters A.M., Asfahani R., Carroll P., Bicknell L., Lescai F., Bright A., Chanudet E., Brooks A., Christou-Savina S., Osman G. (2015). The kinetochore protein, CENPF, is mutated in human ciliopathy and microcephaly phenotypes. J. Med. Genet..

[6] Gilmore E.C., Walsh C.A. (2013). Genetic causes of microcephaly and lessons for neuronal development. Wiley Interdiscip. Rev. Dev. Biol..

[7] Dolk H. (1991). The predictive value of microcephaly during the first year of life for mental retardation at seven years. Dev. Med. Child Neurol..

[8] Morris-Rosendahl D.J., Kaindl A.M. (2015). What next-generation sequencing (NGS) technology has enabled us to learn about primary autosomal recessive microcephaly (MCPH). Mol. Cell. Probes.

[9] Guemez-Gamboa A., Nguyen L.N., Yang H., Zaki M.S., Kara M., Ben-Omran T., Akizu N., Rosti R.O., Rosti B., Scott E. (2015). Inactivating mutations in MFSD2A, required for omega-3 fatty acid transport in brain, cause a lethal microcephaly syndrome. Nat. Genet..

[10] DiStasio A., Driver A., Sund K., Donlin M., Muraleedharan R.M., Pooya S., Kline-Fath B., Kaufman K.M., Prows C.A., Schorry E. (2017). Copb2 is essential for embryogenesis and hypomorphic mutations cause human microcephaly. Hum. Mol. Genet..

[11] Kadir R., Harel T., Markus B., Perez Y., Bakhrat A., Cohen I., Volodarsky M., Feintsein-Linial M., Chervinski E., Zlotogora J. (2016). ALFY-Controlled DVL3 Autophagy Regulates Wnt Signaling, Determining Human Brain Size. PLoS Genet..

[12] Heald R., Khodjakov A. (2015). Thirty years of search and capture: The complex simplicity of mitotic spindle assembly. J. Cell Biol..

[13] Petry S. (2016). Mechanisms of Mitotic Spindle Assembly. Annu. Rev. Biochem..

[14] Prosser S.L., Pelletier L. (2017). Mitotic spindle assembly in animal cells: A fine balancing act. Nat. Rev. Mol. Cell Biol..

[15] Corbett K.D. (2017). Molecular Mechanisms of Spindle Assembly Checkpoint Activation and Silencing. Prog. Mol. Subcell. Biol..

[16] McIntosh J.R. (2017). Assessing the Contributions of Motor Enzymes and Microtubule Dynamics to Mitotic Chromosome Motions. Annu. Rev. Cell Dev. Biol..

[17] Jackson A.P., Eastwood H., Bell S.M., Adu J., Toomes C., Carr I.M., Roberts E., Hampshire D.J., Crow Y.J., Mighell A.J. (2002). Identification of microcephalin, a protein implicated in determining the size of the human brain. Am. J. Hum. Genet..

[18] Trimborn M., Bell S.M., Felix C., Rashid Y., Jafri H., Griffiths P.D., Neumann L.M., Krebs A., Reis A., Sperling K. (2004). Mutations in microcephalin cause aberrant regulation of chromosome condensation. Am. J. Hum. Genet..

[19] Lin S.Y., Rai R., Li K., Xu Z.X., Elledge S.J. (2005). BRIT1/MCPH1 is a DNA damage responsive protein that regulates the Brca1-Chk1 pathway, implicating checkpoint dysfunction in microcephaly. Proc. Natl. Acad. Sci. USA.

[20] Yang Y.J., Baltus A.E., Mathew R.S., Murphy E.A., Evrony G.D., Gonzalez D.M., Wang E.P., Marshall-Walker C.A., Barry B.J., Murn J. (2012). Microcephaly gene links trithorax and REST/NRSF to control neural stem cell proliferation and differentiation. Cell.

[21] Gunster M.J., Satijn D.P., Hamer K.M., den Blaauwen J.L., de Bruijn D., Alkema M.J., van Lohuizen M., van Driel R., Otte A.P. (1997). Identification and characterization of interactions between the vertebrate polycomb-group protein BMI1 and human homologs of polyhomeotic. Mol. Cell. Biol..

[22] Awad S., Al-Dosari M.S., Al-Yacoub N., Colak D., Salih M.A., Alkuraya F.S., Poizat C. (2013). Mutation in PHC1 implicates chromatin remodeling in primary microcephaly pathogenesis. Hum. Mol. Genet..

[23] Schmiesing J.A., Gregson H.C., Zhou S., Yokomori K. (2000). A Human Condensin Complex Containing hCAP-C-hCAP-E and CNAP1, a Homolog of Xenopus XCAP-D2, Colocalizes with Phosphorylated Histone H3 during the Early Stage of Mitotic Chromosome Condensation. Mol. Cell. Biol..

[24] Aono N., Sutani T., Tomonaga T., Mochida S., Yanagida M. (2002). Cnd2 has dual roles in mitotic condensation and interphase. Nature.

[25] Ono T., Losada A., Hirano M., Myers M.P., Neuwald A.F., Hirano T. (2003). Differential contributions of condensin I and condensin II to mitotic chromosome architecture in vertebrate cells. Cell.

[26] Martin C.A., Murray J.E., Carroll P., Leitch A., Mackenzie K.J., Halachev M., Fetit A.E., Keith C., Bicknell L.S., Fluteau A. (2016). Mutations in genes encoding condensin complex proteins cause microcephaly through decatenation failure at mitosis. Genes Dev..

[27] Moynihan L., Jackson A.P., Roberts E., Karbani G., Lewis I., Corry P., Turner G., Mueller R.F., Lench N.J., Woods C.G. (2000). A third novel locus for primary autosomal recessive microcephaly maps to chromosome 9q34. Am. J. Hum. Genet..

[28] Graser S., Stierhof Y.D., Nigg E.A. (2007). Cep68 and Cep215 (Cdk5rap2) are required for centrosome cohesion. J. Cell Sci..

[29] Lizarraga S.B., Margossian S.P., Harris M.H., Campagna D.R., Han A.P., Blevins S., Mudbhary R., Barker J.E., Walsh C.A., Fleming M.D. (2010). Cdk5rap2 regulates centrosome function and chromosome segregation in neuronal progenitors. Development.

[30] Bilgüvar K., Öztürk A.K., Louvi A., Kwan K.Y., Choi M., Tatli B., Yalnizoǧlu D., Tüysüz B., Çaǧlayan A.O., Gökben S. (2010). Whole-exome sequencing identifies recessive WDR62 mutations in severe brain malformations. Nature.

[31] Shohayeb B., Lim N.R., Ho U., Xu Z., Dottori M., Quinn L., Ng D.C.H. (2018). The Role of WD40-Repeat Protein 62 (MCPH2) in Brain Growth: Diverse Molecular and Cellular Mechanisms Required for Cortical Development. Mol. Neurobiol..

[32] Kleylein-Sohn J., Westendorf J., Le Clech M., Habedanck R., Stierhof Y.D., Nigg E.A. (2007). Plk4-Induced Centriole Biogenesis in Human Cells. Dev. Cell.

[33] Hussain M.S., Baig S.M., Neumann S., Nürnberg G., Farooq M., Ahmad I., Alef T., Hennies H.C., Technau M., Altmüller J. (2012). A truncating mutation of CEP135 causes primary microcephaly and disturbed centrosomal function. Am. J. Hum. Genet..

[34] Andersen J.S., Wilkinson C.J., Mayor T., Mortensen P., Nigg E.A., Mann M. (2003). Proteomic characterization of the human centrosome by protein correlation profiling. Nature.

[35] Guernsey D.L., Jiang H., Hussin J., Arnold M., Bouyakdan K., Perry S., Babineau-Sturk T., Beis J., Dumas N., Evans S.C. (2010). Mutations in centrosomal protein CEP152 in primary microcephaly families linked to MCPH4. Am. J. Hum. Genet..

[36] Leidel S., Delattre M., Cerutti L., Baumer K., Gönczy P. (2005). SAS-6 defines a protein family required for centrosome duplication in C. elegans and in human cells. Nat. Cell Biol..

[37] Khan M.A., Rupp V.M., Orpinell M., Hussain M.S., Altmüller J., Steinmetz M.O., Enzinger C., Thiele H., Höhne W., Nürnberg G. (2014). A missense mutation in the PISA domain of HsSAS-6 causes autosomal recessive primary microcephaly in a large consanguineous pakistani family. Hum. Mol. Genet..

[38] Hung L.-Y., Tang C.-J.C., Tang T.K. (2000). Protein 4.1 R-135 Interacts with a Novel Centrosomal Protein (CPAP) Which Is Associated with the gamma -Tubulin Complex. Mol. Cell. Biol..

[39] Leal G.F., Roberts E., Silva E.O., Costa S.M.R., Hampshire D.J., Woods C.G. (2003). A novel locus for autosomal recessive primary microcephaly (MCPH6) maps to 13q12.2. J. Med. Genet..

[40] Bond J., Roberts E., Springell K., Lizarraga S., Scott S., Higgins J., Hampshire D.J., Morrison E.E., Leal G.F., Silva E.O. (2005). A centrosomal mechanism involving CDK5RAP2 and CENPJ controls brain size. Nat. Genet..

[41] Kumar A., Girimaji S.C., Duvvari M.R., Blanton S.H. (2008). Mutations in STIL, encoding a pericentriolar and centrosomal protein, cause primary microcephaly. Am. J. Hum. Genet..

[42] Vulprecht J., David A., Tibelius A., Castiel A., Konotop G., Liu F., Bestvater F., Raab M.S., Zentgraf H., Izraeli S. (2012). STIL is required for centriole duplication in human cells. J. Cell Sci..

[43] Bond J., Roberts E., Mochida G.H., Hampshire D.J., Scott S., Askham J.M., Springell K., Mahadevan M., Crow Y.J., Markham A.F. (2002). ASPM is a major determinant of cerebral cortical size. Nat. Genet..

[44] Fish J.L., Kosodo Y., Enard W., Pääbo S., Huttner W.B. (2006). Aspm specifically maintains symmetric proliferative divisions of neuroepithelial cells. Proc. Natl. Acad. Sci. USA.

[45] Létard P., Drunat S., Vial Y., Duerinckx S., Ernault A., Amram D., Arpin S., Bertoli M., Busa T., Ceulemans B. (2018). Autosomal recessive primary microcephaly due to ASPM mutations: An update. Hum. Mutat..

[46] Di Cunto F., Calautti E., Hsiao J., Ong L., Topley G., Turco E., Dotto G.P. (1998). Citron Rho-interacting kinase, a novel tissue-specific Ser/Thr kinase encompassing the Rho-Rac-binding protein citron. J. Biol. Chem..

[47] Li H., Bielas S.L., Zaki M.S., Ismail S., Farfara D., Um K., Rosti R.O., Scott E.C., Tu S., Chi N.C. (2016). Biallelic Mutations in Citron Kinase Link Mitotic Cytokinesis to Human Primary Microcephaly. Am. J. Hum. Genet..

[48] Gruneberg U., Neef R., Li X., Chan E.H.Y., Chalamalasetty R.B., Nigg E.A., Barr F.A. (2006). KIF14 and citron kinase act together to promote efficient cytokinesis. J. Cell Biol..

[49] Moawia A., Shaheen R., Rasool S., Waseem S.S., Ewida N., Budde B., Kawalia A., Motameny S., Khan K., Fatima A. (2017). Mutations of KIF14 cause primary microcephaly by impairing cytokinesis. Ann. Neurol..

[50] Perez Y., Bar-Yaacov R., Kadir R., Wormser O., Shelef I., Birk O.S., Flusser H., Birnbaum R.Y. (2019). Mutations in the microtubule-associated protein MAP11 (C7orf43) cause microcephaly in humans and zebrafish. Brain.

[51] Genin A., Desir J., Lambert N., Biervliet M., Van der Aa N., Pierquin G., Killian A., Tosi M., Urbina M., Lefort A. (2012). Kinetochore KMN network gene CASC5 mutated in primary microcephaly. Hum. Mol. Genet..

[52] Yen T.J., Li G., Schaar B.T., Szilak I., Cleveland D.W. (1992). CENP-E is a putative kinetochore motor that accumulates just before mitosis. Nature.

[53] Mirzaa G.M., Vitre B., Carpenter G., Abramowicz I., Gleeson J.G., Paciorkowski A.R., Cleveland D.W., Dobyns W.B., O’Driscoll M. (2014). Mutations in CENPE define a novel kinetochore-centromeric mechanism for microcephalic primordial dwarfism. Hum. Genet..

[54] Meyerson M., Harlow E. (1994). Identification of G1 kinase activity for cdk6, a novel cyclin D partner. Mol. Cell. Biol..

[55] Hussain M.S., Baig S.M., Neumann S., Peche V.S., Szczepanski S., Nürnberg G., Tariq M., Jameel M., Khan T.N., Fatima A. (2013). CDK6 associates with the centrosome during mitosis and is mutated in a large pakistani family with primary microcephaly. Hum. Mol. Genet..

[56] Asencio C., Davidson I.F., Santarella-Mellwig R., Ly-Hartig T.B.N., Mall M., Wallenfang M.R., Mattaj I.W., Gorjánácz M. (2012). Coordination of kinase and phosphatase activities by Lem4 enables nuclear envelope reassembly during mitosis. Cell.

[57] Shaheen R., Maddirevula S., Ewida N., Alsahli S., Abdel-Salam G.M.H., Zaki M.S., Al Tala S., Alhashem A., Softah A., Al-Owain M. (2019). Genomic and phenotypic delineation of congenital microcephaly. Genet. Med..

[58] Clausen T.H., Lamark T., Isakson P., Finley K., Larsen K.B., Brech A., Øvervatn A., Stenmark H., Bjørkøy G., Simonsen A. (2010). p62/SQSTM1 and ALFY interact to facilitate the formation of p62 bodies/ALIS and their degradation by autophagy. Autophagy.

[59] Harrison-Lavoie K.J., Lewis V.A., Hynes G.M., Collison K.S., Nutland E., Willison K.R. (1993). A 102 kDa subunit of a Golgi-associated particle has homology to beta subunits of trimeric G proteins. EMBO J..

[60] Angers M., Uldry M., Kong D., Gimble J.M., Jetten A.M. (2008). Mfsd2a encodes a novel major facilitator superfamily domain-containing protein highly induced in brown adipose tissue during fasting and adaptive thermogenesis. Biochem. J..

[61] Marthiens V., Rujano M.A., Pennetier C., Tessier S., Paul-Gilloteaux P., Basto R. (2013). Centrosome amplification causes microcephaly. Nat. Cell Biol..

[62] Nano M., Basto R. (2017). Consequences of centrosome dysfunction during brain development. Advances in Experimental Medicine and Biology.

[63] Maiato H., Logarinho E. (2014). Mitotic spindle multipolarity without centrosome amplification. Nat. Cell Biol..

[64] Paronett E.M., Meechan D.W., Karpinski B.A., LaMantia A.-S., Maynard T.M. (2015). Ranbp1, Deleted in DiGeorge/22q11.2 Deletion Syndrome, is a Microcephaly Gene That Selectively Disrupts Layer 2/3 Cortical Projection Neuron Generation. Cereb. Cortex.

[65] Di Fiore B., Ciciarello M., Mangiacasale R., Palena A., Tassin A.-M., Cundari E., Lavia P. (2003). Mammalian RanBP1 regulates centrosome cohesion during mitosis. J. Cell Sci..

[66] Lavia P. (2016). The GTPase RAN regulates multiple steps of the centrosome life cycle. Chromosome Res..

[67] Saade M., Blanco-Ameijeiras J., Gonzalez-Gobartt E., Martí E. (2018). A centrosomal view of CNS growth. Development.

[68] Basit S., Al-Harbi K.M., Alhijji S.A.M., Albalawi A.M., Alharby E., Eldardear A., Samman M.I. (2016). CIT, a gene involved in neurogenic cytokinesis, is mutated in human primary microcephaly. Hum. Genet..

[69] Harding B.N., Moccia A., Drunat S., Soukarieh O., Tubeuf H., Chitty L.S., Verloes A., Gressens P., El Ghouzzi V., Joriot S. (2016). Mutations in Citron Kinase Cause Recessive Microlissencephaly with Multinucleated Neurons. Am. J. Hum. Genet..

[70] Shaheen R., Hashem A., Abdel-Salam G.M.H., Al-Fadhli F., Ewida N., Alkuraya F.S. (2016). Mutations in CIT, encoding citron rho-interacting serine/threonine kinase, cause severe primary microcephaly in humans. Hum. Genet..

[71] Bianchi F.T., Tocco C., Pallavicini G., Liu Y., Vernì F., Merigliano C., Bonaccorsi S., El-Assawy N., Priano L., Gai M. (2017). Citron Kinase Deficiency Leads to Chromosomal Instability and TP53-Sensitive Microcephaly. Cell Rep..

[72] Gai M., Bianchi F.T., Vagnoni C., Vernì F., Bonaccorsi S., Pasquero S., Berto G.E., Sgrò F., Chiotto A.A., Annaratone L. (2017). ASPM and CITK regulate spindle orientation by affecting the dynamics of astral microtubules. EMBO Rep..

[73] Bianchi F.T., Gai M., Berto G.E., Di Cunto F. (2017). Of rings and spines: The multiple facets of Citron proteins in neural development. Small GTPases.

[74] D’Avino P.P. (2017). Citron kinase—Renaissance of a neglected mitotic kinase. J. Cell Sci..

[75] Ferreira R.O., Garcez P.P. (2019). Dissecting the Toxic Effects of Zika Virus Proteins on Neural Progenitor Cells. Neuron.

[76] Matsuura S., Matsumoto Y., Morishima K., Izumi H., Matsumoto H., Ito E., Tsutsui K., Kobayashi J., Tauchi H., Kajiwara Y. (2006). MonoallelicBUB1B mutations and defective mitotic-spindle checkpoint in seven families with premature chromatid separation (PCS) syndrome. Am. J. Med. Genet. Part A.

[77] de Wolf B., Kops G.J.P.L. (2017). Kinetochore malfunction in human pathologies. Advances in Experimental Medicine and Biology.

[78] Taylor A.M., Shih J., Ha G., Gao G.F., Zhang X., Berger A.C., Schumacher S.E., Wang C., Hu H., Liu J. (2018). Genomic and Functional Approaches to Understanding Cancer Aneuploidy. Cancer Cell.

[79] Hara M., Fukagawa T. (2018). Kinetochore assembly and disassembly during mitotic entry and exit. Curr. Opin. Cell Biol..

[80] Vukušić K., Buđa R., Tolić I.M. (2019). Force-generating mechanisms of anaphase in human cells. J. Cell Sci..

[81] Cimini D., Degrassi F. (2005). Aneuploidy: A matter of bad connections. Trends Cell Biol..

[82] Gregan J., Polakova S., Zhang L., Tolić-Nørrelykke I.M., Cimini D. (2011). Merotelic kinetochore attachment: Causes and effects. Trends Cell Biol..

[83] Musacchio A., Salmon E.D. (2007). The spindle-assembly checkpoint in space and time. Nat. Rev. Mol. Cell Biol..

[84] Etemad B., Kops G.J.P.L. (2016). Attachment issues: Kinetochore transformations and spindle checkpoint silencing. Curr. Opin. Cell Biol..

[85] Foley E.A., Kapoor T.M. (2013). Microtubule attachment and spindle assembly checkpoint signalling at the kinetochore. Nat. Rev. Mol. Cell Biol..

[86] Musacchio A. (2015). The Molecular Biology of Spindle Assembly Checkpoint Signaling Dynamics. Curr. Biol..

[87] Dong Y., Vanden Beldt K.J., Meng X., Khodjakov A., McEwen B.F. (2007). The outer plate in vertebrate kinetochores is a flexible network with multiple microtubule interactions. Nat. Cell Biol..

[88] Lampson M.A., Cheeseman I.M. (2011). Sensing centromere tension: Aurora B and the regulation of kinetochore function. Trends Cell Biol..

[89] Hauf S., Cole R.W., LaTerra S., Zimmer C., Schnapp G., Walter R., Heckel A., van Meel J., Rieder C.L., Peters J.-M. (2003). The small molecule Hesperadin reveals a role for Aurora B in correcting kinetochore-microtubule attachment and in maintaining the spindle assembly checkpoint. J. Cell Biol..

[90] McKinley K.L., Cheeseman I.M. (2016). The molecular basis for centromere identity and function. Nat. Rev. Mol. Cell Biol..

[91] Pesenti M.E., Weir J.R., Musacchio A. (2016). Progress in the structural and functional characterization of kinetochores. Curr. Opin. Struct. Biol..

[92] Saurin A.T. (2018). Kinase and phosphatase cross-talk at the kinetochore. Front. Cell Dev. Biol..

[93] Jamieson C.R., Govaerts C., Abramowicz M.J. (1999). Primary autosomal recessive microcephaly: Homozygosity mapping of MCPH4 to chromosome 15. Am. J. Hum. Genet..

[94] Saadi A., Verny F., Siquier-Pernet K., Bole-Feysot C., Nitschke P., Munnich A., Abada-Dendib M., Chaouch M., Abramowicz M., Colleaux L. (2016). Refining the phenotype associated with CASC5 mutation. Neurogenetics.

[95] Szczepanski S., Hussain M.S., Sur I., Altmüller J., Thiele H., Abdullah U., Waseem S.S., Moawia A., Nürnberg G., Noegel A.A. (2016). A novel homozygous splicing mutation of CASC5 causes primary microcephaly in a large Pakistani family. Hum. Genet..

[96] Zarate Y.A., Kaylor J.A., Bosanko K., Lau S., Vargas J., Gao H. (2016). First clinical report of an infant with microcephaly and CASC5 mutations. Am. J. Med. Genet. Part A.

[97] Caldas G.V., Deluca J.G. (2014). KNL1: Bringing order to the kinetochore. Chromosoma.

[98] Shi L., Qalieh A., Lam M.M., Keil J.M., Kwan K.Y. (2019). Robust elimination of genome-damaged cells safeguards against brain somatic aneuploidy following Knl1 deletion. Nat. Commun..

[99] Janssen A., Van Der Burg M., Szuhai K., Kops G.J.P.L., Medema R.H. (2011). Chromosome segregation errors as a cause of DNA damage and structural chromosome aberrations. Science.

[100] Crasta K., Ganem N.J., Dagher R., Lantermann A.B., Ivanova E.V., Pan Y., Nezi L., Protopopov A., Chowdhury D., Pellman D. (2012). DNA breaks and chromosome pulverization from errors in mitosis. Nature.

[101] Santaguida S., Richardson A., Iyer D.R., M’Saad O., Zasadil L., Knouse K.A., Wong Y.L., Rhind N., Desai A., Amon A. (2017). Chromosome Mis-segregation Generates Cell-Cycle-Arrested Cells with Complex Karyotypes that Are Eliminated by the Immune System. Dev. Cell.

[102] Omer Javed A., Li Y., Muffat J., Su K.C., Cohen M.A., Lungjangwa T., Aubourg P., Cheeseman I.M., Jaenisch R. (2018). Microcephaly Modeling of Kinetochore Mutation Reveals a Brain-Specific Phenotype. Cell Rep..

[103] Suijkerbuijk S.J.E., Van Osch M.H.J., Bos F.L., Hanks S., Rahman N., Kops G.J.P.L. (2010). Molecular causes for BUBR1 dysfunction in the human cancer predisposition syndrome mosaic variegated aneuploidy. Cancer Res..

[104] Overlack K., Primorac I., Vleugel M., Krenn V., Maffini S., Hoffmann I., Kops G.J.P.L., Musacchio A. (2015). A molecular basis for the differential roles of Bubl and BubR1 in the spindle assembly checkpoint. eLife.

[105] Ciossani G., Overlack K., Petrovic A., Huis In ’T Veld P.J., Koerner C., Wohlgemuth S., Maffini S., Musacchio A. (2018). The kinetochore proteins CENP-E and CENP-F directly and specifically interact with distinct BUB mitotic checkpoint Ser/Thr kinases. J. Biol. Chem..

[106] Huang Y., Lin L., Liu X., Ye S., Yao P.Y., Wang W., Yang F., Gao X., Li J., Zhang Y. (2019). BubR1 phosphorylates CENP-E as a switch enabling the transition from lateral association to end-on capture of spindle microtubules. Cell Res..

[107] Suijkerbuijk S.J.E., Vleugel M., Teixeira A., Kops G.J.P.L. (2012). Integration of Kinase and Phosphatase Activities by BUBR1 Ensures Formation of Stable Kinetochore-Microtubule Attachments. Dev. Cell.

[108] Simmons A.J., Park R., Sterling N.A., Jang M.H., Van Deursen J.M.A., Yen T.J., Cho S.H., Kim S. (2019). Nearly complete deletion of BubR1 causes microcephaly through shortened mitosis and massive cell death. Hum. Mol. Genet..

[109] Snape K., Hanks S., Ruark E., Barros-Núñez P., Elliott A., Murray A., Lane A.H., Shannon N., Callier P., Chitayat D. (2011). Mutations in CEP57 cause mosaic variegated aneuploidy syndrome. Nat. Genet..

[110] Zhou H., Wang T., Zheng T., Teng J., Chen J. (2016). Cep57 is a Mis12-interacting kinetochore protein involved in kinetochore targeting of Mad1-Mad2. Nat. Commun..

[111] Yost S., De Wolf B., Hanks S., Zachariou A., Marcozzi C., Clarke M., De Voer R.M., Etemad B., Uijttewaal E., Ramsay E. (2017). Biallelic TRIP13 mutations predispose to Wilms tumor and chromosome missegregation. Nat. Genet..

[112] Yao X., Abrieu A., Zheng Y., Sullivan K.F., Cleveland D.W. (2000). CENP-E forms a link between attachment of spindle microtubules to kinetochores and the mitotic checkpoint. Nat. Cell Biol..

[113] Weaver B.A.A., Bonday Z.Q., Putkey F.R., Kops G.J.P.L., Silk A.D., Cleveland D.W. (2003). Centromere-associated protein-E is essential for the mammalian mitotic checkpoint to prevent aneuploidy due to single chromosome loss. J. Cell Biol..

[114] Guo Y., Kim C., Ahmad S., Zhang J., Mao Y. (2012). CENP-E--dependent BubR1 autophosphorylation enhances chromosome alignment and the mitotic checkpoint. J. Cell Biol..

[115] Taveras C., Liu C., Mao Y. (2019). A tension-independent mechanism reduces Aurora B-mediated phosphorylation upon microtubule capture by CENP-E at the kinetochore. Cell Cycle.

[116] Weaver B.A.A., Silk A.D., Montagna C., Verdier-Pinard P., Cleveland D.W. (2007). Aneuploidy Acts Both Oncogenically and as a Tumor Suppressor. Cancer Cell.

[117] Varis A., Salmela A.L., Kallio M.J. (2006). Cenp-F (mitosin) is more than a mitotic marker. Chromosoma.

[118] Liao H., Winkfein R.J., Mack G., Rattner J.B., Yen T.J. (1995). CENP-F is a protein of the nuclear matrix that assembles onto kinetochores at late G2 and is rapidly degraded after mitosis. J. Cell Biol..

[119] Zhu X., Chang K.H., He D., Mancini M.A., Brinkley W.R., Lee W.H. (1995). The C terminus of mitosin is essential for its nuclear localization, centromere/kinetochore targeting, and dimerization. J. Biol. Chem..

[120] Gurden M.D.J., Holland A.J., Van Zon W., Tighe A., Vergnolle M.A., Andres D.A., Spielmann H.P., Malumbres M., Wolthuis R.M.F., Cleveland D.W. (2010). Cdc20 is required for the post-anaphase, KEN-dependent degradation of centromere protein F. J. Cell Sci..

[121] Feng J., Huang H., Yen T.J. (2006). CENP-F is a novel microtubule-binding protein that is essential for kinetochore attachments and affects the duration of the mitotic checkpoint delay. Chromosoma.

[122] Yang Z.Y., Guo J., Li N., Qian M., Wang S.N., Zhu X.L. (2003). Mitosin/CENP-F is a conserved kinetochore protein subjected to cytoplasmic dynein-mediated poleward transport. Cell Res..

[123] Vergnolle M.A.S., Taylor S.S. (2007). Cenp-F Links Kinetochores to Ndel1/Nde1/Lis1/Dynein Microtubule Motor Complexes. Curr. Biol..

[124] Bolhy S., Bouhlel I., Dultz E., Nayak T., Zuccolo M., Gatti X., Vallee R., Ellenberg J., Doye V. (2011). A Nup133-dependent NPC-anchored network tethers centrosomes to the nuclear envelope in prophase. J. Cell Biol..

[125] Berto A., Doye V. (2018). Regulation of Cenp-F localization to nuclear pores and kinetochores. Cell Cycle.

[126] Zuccolo M., Alves A., Galy V., Bolhy S., Formstecher E., Racine V., Sibarita J.B., Fukagawa T., Shiekhattar R., Yen T. (2007). The human Nup107-160 nuclear pore subcomplex contributes to proper kinetochore functions. EMBO J..

[127] Filges I., Bruder E., Brandal K., Meier S., Undlien D.E., Waage T.R., Hoesli I., Schubach M., de Beer T., Sheng Y. (2016). Strømme Syndrome Is a Ciliary Disorder Caused by Mutations in CENPF. Hum. Mutat..

[128] Ozkinay F., Atik T., Isik E., Gormez Z., Sagiroglu M., Sahin O.A., Corduk N., Onay H. (2017). A further family of Stromme syndrome carrying CENPF mutation. Am. J. Med. Genet. A.

[129] Vasu S., Shah S., Orjalo A., Park M., Fischer W.H., Forbes D.J. (2001). Novel vertebrate nucleoporins Nup133 and Nup160 play a role in mRNA export. J. Cell Biol..

[130] Hu D.J.K., Baffet A.D., Nayak T., Akhmanova A., Doye V., Vallee R.B. (2013). XDynein recruitment to nuclear pores activates apical nuclear migration and mitotic entry in brain progenitor cells. Cell.

[131] Splinter D., Tanenbaum M.E., Lindqvist A., Jaarsma D., Flotho A., Yu K.L., Grigoriev I., Engelsma D., Haasdijk E.D., Keijzer N. (2010). Bicaudal D2, Dynein, and Kinesin-1 Associate with Nuclear Pore Complexes and Regulate Centrosome and Nuclear Positioning during Mitotic Entry. PLoS Biol..

[132] Fujita A., Tsukaguchi H., Koshimizu E., Nakazato H., Itoh K., Kuraoka S., Komohara Y., Shiina M., Nakamura S., Kitajima M. (2018). Homozygous splicing mutation in NUP133 causes Galloway-Mowat syndrome. Ann. Neurol..

[133] Rosti R.O., Sotak B.N., Bielas S.L., Bhat G., Silhavy J.L., Aslanger A.D., Altunoglu U., Bilge I., Tasdemir M., Yzaguirrem A.D. (2017). Homozygous mutation in NUP107 leads to microcephaly with steroid-resistant nephrotic condition similar to Galloway-Mowat syndrome. J. Med. Genet..

[134] Gogendeau D., Siudeja K., Gambarotto D., Pennetier C., Bardin A.J., Basto R. (2015). Aneuploidy causes premature differentiation of neural and intestinal stem cells. Nat. Commun..

[135] Poulton J.S., Cuningham J.C., Peifer M. (2017). Centrosome and spindle assembly checkpoint loss leads to neural apoptosis and reduced brain size. J. Cell Biol..

[136] Quadrato G., Arlotta P. (2017). Present and future of modeling human brain development in 3D organoids. Curr. Opin. Cell Biol..

[137] Trujillo C.A., Muotri A.R. (2018). Brain Organoids and the Study of Neurodevelopment. Trends Mol. Med..

